# Incidence of pediatric delirium and withdrawal outside the PICU: findings from a pediatric cardiology ward

**DOI:** 10.1007/s00431-025-06395-z

**Published:** 2025-08-21

**Authors:** Alexander Simma, Susanne Haase, Ines Ritthaler, Juliane Engel, Johannes Nordmeyer, Felix Neunhoeffer

**Affiliations:** Department of Pediatric Cardiology, Pulmonology and Pediatric Intensive Care Medicine, University Children’s Hospital Tübingen, University of Tübingen, Hoppe-Seyler-Str. 1, 72076 Tübingen, Germany

**Keywords:** Pediatric delirium, Iatrogenic withdrawal syndrome, Pediatric cardiology

## Abstract

Pediatric delirium, a severe acute brain dysfunction, is associated with increased mortality, morbidity, and prolonged hospital stays. Despite its substantial impact, it often goes unrecognized and is not routinely screened for, resulting in underrecognition. Delirium is common in pediatric intensive care units (PICUs), with a prevalence of 34% in a recent meta-analysis. However, data on its occurrence outside the PICU on general pediatric wards are limited. The objective of this study was to determine the incidence of delirium and withdrawal in hospitalized children and adolescents in a pediatric cardiology ward. We conducted a prospective observational study at a tertiary referral center, including all admissions between January 1 and December 31, 2022. Pediatric delirium and withdrawal were screened using the validated Sophia Observation Scale–Pediatric Delirium (SOS-PD), as part of a nurse-driven screening protocol. We evaluated 596 continuous admissions and found that 94 (15.8%) were affected by pediatric delirium, withdrawal, or both. A positive SOS-PD score was associated with an increased length of hospital stay (13 days vs. 3 days; *p* < 0.001) and treatment in our PICU (89.4% vs. 24.1%, *p* < 0.001). Patients with a positive SOS-PD score were also younger (0.5 years vs. 3 years, *p* < 0.001), and most were suffering from congenital heart disease (89.4% vs. 51%; *p* < 0.001).

*Conclusion*: Delirium and withdrawal occur frequently outside the PICU, affecting more than one in six patients in a pediatric cardiology ward. A nurse-driven screening using the SOS-PD can identify affected patients and possible risk factors.

**Table:** 

**What is Known:** *• Pediatric delirium is a severe acute brain dysfunction associated with increased mortality, morbidity, and prolonged hospital stays.* *• Despite its clinical relevance, pediatric delirium is often under-recognized and rarely screened for outside pediatric intensive care units (PICUs).* **What is New:** *• Delirium and withdrawal occur frequently outside the PICU, affecting over one in six patients in a pediatric cardiology ward.* *• Nurse-driven screening using the SOS-PD is feasible for identifying affected patients on general pediatric wards.*

**What is Known:**

*• Pediatric delirium is a severe acute brain dysfunction associated with increased mortality, morbidity, and prolonged hospital stays.*

*• Despite its clinical relevance, pediatric delirium is often under-recognized and rarely screened for outside pediatric intensive care units (PICUs).*

**What is New:**

*• Delirium and withdrawal occur frequently outside the PICU, affecting over one in six patients in a pediatric cardiology ward.*

*• Nurse-driven screening using the SOS-PD is feasible for identifying affected patients on general pediatric wards.*

## Introduction

Pediatric delirium is a severe and acute brain dysfunction associated with increased mortality, morbidity, and prolonged hospital stays [1]. It is driven by a combination of pre-existing risk factors such as disease severity, need for mechanical ventilation, vasoactive drugs, young age, and developmental delay [1, 2]. More importantly, there are also potentially modifiable risk factors in iatrogenic withdrawal syndrome, the use of benzodiazepines, opiates, steroids, and physical restraints [1, 2].

Delirium is common in pediatric intensive care units (PICUs), with a reported prevalence of 34% [3]. More than a quarter of these patients are reported to experience a recurring episode of delirium according to a recent meta-analysis [3]. Given the high prevalence, several validated assessment tools have been developed to detect this condition [4–7]. Routine screening and monitoring for pediatric delirium are recommended by a variety of international clinical practice guidelines [8]. These validated assessment tools demonstrate high concordance with psychiatric evaluation, high sensitivity and specificity, and are easy to perform, enabling a nurse-driven routine screening [1, 9, 10]. The Sophia Observation Scale–Pediatric Delirium (SOS-PD) consists of two parts: the Sophia Observation Scale (SOS) for withdrawal and the pediatric delirium (PD) scale. This tool stands out as the distinctive score that separates delirium from withdrawal [6].

Routine screening is essential not only for timely detection but also for prevention and management, including non-pharmacological interventions [1, 11, 12]. Nevertheless, in most PICUs, delirium is not routinely screened for and often goes unrecognized, leading to its underrecognition [13, 14].

In contrast, literature on pediatric delirium outside the PICU is scarce. Existing studies focus on hematology, oncology, and bone marrow transplant settings [15]. Notably, no data are currently available on pediatric delirium in cardiology wards, despite elevated risk in young children, especially after cardiac bypass surgery [1, 3, 16].

This observational study aims to determine delirium and withdrawal outside the PICU by implementing a nurse-driven screening using the SOS-PD and to identify associated patient characteristics and potential risk factors.

## Methods

### Study design

This is a single-center, observational, prospective study. Over 2 months (November–December 2021), we trained the multiprofessional team from a 14-bed pediatric cardiology ward in the structured detection of pediatric delirium and withdrawal using the SOS-PD. Subsequently, the study spanned January 1 to December 31, 2022, with only new staff receiving additional training.

### Nurse-driven screening

Our pediatric hospital is a tertiary referral center with a mixed cardiac, surgical, and general PICU. In our pediatric cardiology ward, we treat patients with congenital heart disease (CHD) both pre- and postoperatively, as well as patients undergoing cardiac catheter intervention for various indications. Depending on demand, general pediatric cases are also sometimes admitted to this ward.

All consecutive admissions to our pediatric cardiology ward from January 1 st to December 31 st were eligible. Patients with a hospital stay of fewer than 24 h and those over the age of 18 years at the time of admission were excluded.

Clinical data from all included individuals contains age at admission, sex, length of stay, diagnoses, and SOS-PD results. Data on analgesia and sedation were collected based on predefined medication variables in the case report form. These included non-opioid analgesics like paracetamol, metamizole, and ibuprofen; opioid analgesics like morphine and fentanyl; and sedatives and adjunctive agents including midazolam, clonidine, esketamine, chloralhydrate, levomepromazine, and melatonin. All medications refer specifically to those administered during the stay on the pediatric cardiology ward, regardless of prior PICU treatment.

Data were collected from our digital patient data management systems, Meona® (Mesalvo Freiburg GmbH, Freiburg, Germany) and i.s.h. Med (Cerner Health Services Deutschland GmbH, Berlin, Germany).

### Delirium and withdrawal screening (SOS-PD)

In the SOS-PD, we used a validated tool to screen pediatric patients regularly for delirium and withdrawal [6, 8]. Originally developed for use in the PICU, the SOS-PD combines 23 items related to autonomic dysfunction, central nervous system irritability, gastrointestinal symptoms, and parental observations. Because clinical features of withdrawal and delirium often overlap, the tool allows for scoring that may reflect either or both conditions. A score > 3 on the SOS subscale indicates withdrawal, while a score > 3 on the PD subscale suggests delirium [6]. Consistent with our PICU standard, the SOS-PD score was routinely conducted toward the end of each nurse’s shift, resulting in three assessments per day: early, late, and night shift, respectively [12].

#### Diagnosis classification

To enable subgroup analysis, each patient was assigned a single main diagnosis based on their ICD-10-GM codes. Diagnoses were grouped into clinically relevant categories using a hierarchical, prefix-based mapping system. Categories included congenital heart disease, acquired cardiac disease, and other organ system-based groups. Administrative codes were excluded, and uncategorized diagnoses were manually reviewed. Remaining cases were grouped under “Other.”

### Statistical analysis

Study data were collected and managed using REDCap (Research Electronic Data Capture), hosted at the University of Tübingen. Descriptive statistics were calculated for the total cohort and stratified by SOS-PD status. Categorical variables were reported as frequencies and percentages, while continuous variables were presented as means with standard deviations (SD) and medians with interquartile ranges (IQR), depending on distribution. Group comparisons between patients screening positive and negative for SOS-PD were performed. For continuous variables, the Wilcoxon rank-sum test was used due to non-normal distributions. For categorical variables, chi-squared tests were applied. All *p* values were obtained from two-sided tests with a threshold for statistically significant results at a 0.05 alpha level. All statistical analyses were conducted using R, version 4.5.0 (R Foundation for Statistical Computing, Vienna, Austria).

### Ethics statement

The study “Incidence of pediatric delirium and withdrawal outside the PICU: findings from a pediatric cardiology ward” was approved by the Ethics Committee of the University of Tübingen on 13 October 2021 (481/2021BO1). The procedures followed were in accordance with the Helsinki Declaration of 1975. Written informed consent was not required.

## Results

### Study population

We included 596 consecutive admissions from 448 individual patients for our analysis. In all included patients (*n* = 596), at least one SOS-PD score was obtained. We included a total of 8612 score results obtained on 3505 days, yielding an average of 2.5 scoring results per patient-day on the pediatric cardiology ward. The obtained scoring results were distributed equally between the shifts, with no significant difference in the frequency of delirium or withdrawal observed during night shifts.

Of these 596 patients, 356 (59.7%) were male. The median age at admission was 1.8 years (IQR 7.1 years). The median length of hospital stay was 4 days (IQR 8 days), with a PICU admission reported in 205 patients (34.4%) (Table 1). Most patients from this pediatric cardiology ward had a congenital heart disease (*n* = 340, 57%) or an acquired heart disease (*n* = 139, 23.3%) as the main reason for their admission.

**Table 1:** Characteristics of the study population

Characteristics	General population (*n* = 596) Number (%)	Patients with delirium (PD = positive) (*n* = 81) Number (%)	Patients with withdrawal (SOS = positive) (*n* = 78) Number (%)	Patients without delirium or withdrawal (SOS-PD negative)
Gender				
Male	356 (59.7)	43 (53.1)	42 (53.8)	306 (61)
Age on admission (years)^a^	1.81 (0.37–7.43)	0.45 (0.25–0.94)	0.45 (0.26–0.91)	2.94 (0.49–9.25)
Patients with a PICU admission	205 (34.4)	75 (92.6)	70 (89.7)	121 (24.1)
Length of PICU stay (days)^a^	5 (3–13)	5 (3–13)	6 (4–18)	4 (2–12)
Length of hospital stay (days)^a^	4 (2–10)	13 (9–28)	13.5 (9–30)	3 (2–7)
Congenital heart disease	340 (57)	72 (88.9)	68 (87.2)	256 (51)

^a^Median (IQR)

We found that 81 (13.6%) patients scored positive for delirium and 78 (13.1%) for withdrawal. There is a major overlap between those two groups, with 65 (10.9%) patients scoring positive for both delirium and withdrawal (Table 1). This corresponds to 16 patients who screened positive for delirium only and 13 patients who screened positive for withdrawal only. In total, 94 (15.8%) cases had any positive SOS-PD results.

### SOS-PD positive population

Patients with any positive SOS-PD score (*n* = 94, 15.8%) for delirium, withdrawal, or both showed distinct characteristics compared to those with a negative SOS-PD score (*n* = 502, 84.2%) (Table 2).

**Table 2:** Association between population characteristics and the SOS-PD score result

Characteristics	Patients with positive SOS-PD score (*n* = 94) Number (%)	Patients with negative SOS-PD score (*n* = 502) Number (%)	*p* value
Gender			
Male	50 (53.2)	306 (61)	0.16
Age on admission (years)^a^	0.46 (0.25–0.96)	2.94 (0.49–9.25)	< 0.001
Stay on PICU	84 (89.4)	121 (24.1)	< 0.001
Length of PICU stay (days)^a^	5 (3–13)	4 (2–12)	0.012
Length of hospital stay (days)^a^	13 (9–25)	3.0 (2–7)	< 0.001
Diagnoses			
CHD	84 (89.4)	256 (51.0)	< 0.001
Acquired cardiac disease	7 (7.4)	122 (24.3)	< 0.001
Respiratory disease	0 (0)	37 (7.4%)	0.013
Other^b^	3 (3.2)	87 (17.3)	
Medication received^c^			
Paracetamol	87 (92.6)	200 (39.8)	< 0.001
Metamizole	37 (39.4)	71 (14.1)	< 0.001
Ibuprofen	53 (56.4)	134 (26.7)	< 0.001
Morphine	42 (44.7)	15 (3)	< 0.001
Fentanyl	12 (12.8)	20 (4)	< 0.001
Esketamine	18 (19.1)	33 (6.6)	< 0.001
Midazolam	27 (28.7)	45 (9)	< 0.001
Clonidine	47 (50)	22 (4.4)	< 0.001
Chloral hydrate	65 (69.1)	59 (11.8)	< 0.001
Levomepromazine	25 (26.6)	17 (3.4)	< 0.001
Melatonin	22 (23.4)	18 (3.6)	< 0.001

^a^Median (IQR)

^b^Including infectious disease, neurology and neurosurgery, trauma, gastrointestinal disease, hematology and oncology, ENT, endocrinology, and other not classified diagnoses

^c^Medication received on the pediatric cardiology ward

### Demographics and length of hospital stay

**Fig. 1:**
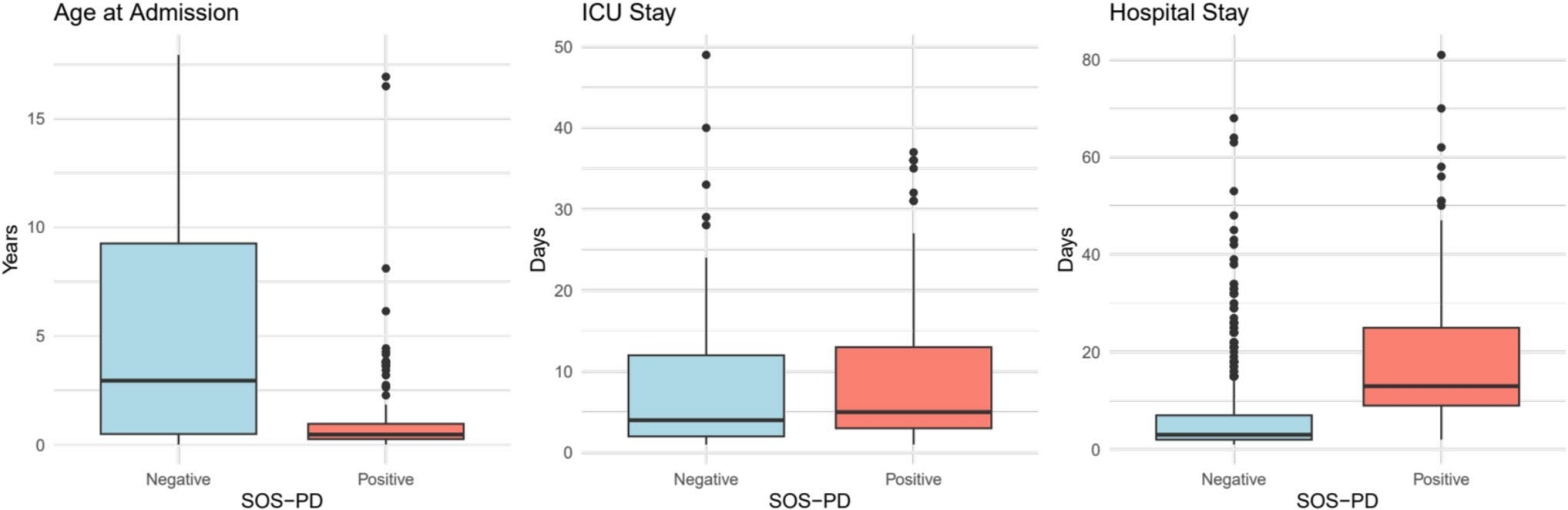
Association between age on admission, length of PICU stay, length of hospital stay, and SOS-PD result

Patients with any positive SOS-PD were significantly younger on admission (median 0.46 years, IQR 2.7 years, IQR 0.8 years) compared to those with a negative score (median age 2.94 years, IQR 8.76 years, *p* < 0.001) (Fig. 1). There was no significant difference in gender distribution between groups (53.2% male vs. 61% male; *p* = 0.16). PICU treatment was much more common in the SOS-PD positive group (89.4% vs. 24.1%; *p* < 0.001). There was a small but significant difference in the length of stay in the PICU (median 5 days, IQR 10 days vs. median 4 days, IQR 10 days; *p* = 0.012). Furthermore, patients with any positive SOS-PD score had a substantially longer overall length of hospital stay (median 13 days, IQR 16 days vs. median 3 days, IQR 5 days; *p* < 0.001) (Fig. 1).

### Medications received

Patients with any positive SOS-PD score received more non-opioid analgesics such as paracetamol (92.6% vs. 39.8%; *p* < 0.001), metamizole (39.4% vs. 14.1%; *p* < 0.001), and ibuprofen (56.4% vs. 26.2%; *p* < 0.001) (Table 1). They also received more opioid analgesics in morphine (44.7% vs. 3%; *p* < 0.001) or fentanyl (12.8% vs. 4%; *p* < 0.001) as continuous intravenous application. Only very few or none received tilidine (*n* = 17, 2.9%), piritramide (*n* = 6, 1%), tramadol (*n* = 2. 0.3%), or hydromorphone (*n* = 0).

Furthermore, patients with any positive score also receive more adjunctive sedative agents such as chloral hydrate (69.1% vs. 11.8%; *p* < 0.001), clonidine (50% vs. 4.4%; *p* < 0.001), levomepromazine (26.6% vs. 3.4%; *p* < 0.001), or melatonin (23.4% vs. 3.6%; *p* < 0.001).

Esketamine (19.1% vs. 6.6%; *p* < 0.01) and midazolam (28.7% vs. 9%; *p* < 0.01) have mostly been used for interventional anesthesia for the removal of chest tubes or pacemaker wires, for instance. Two patients received midazolam as a continuous intravenous application, both of whom scored positive for delirium and withdrawal.

## Discussion

This prospective observational cohort study is the first to show that pediatric delirium and withdrawal occur frequently in a pediatric cardiology ward setting, affecting more than one in six pediatric patients over 12 months. Furthermore, nurse-driven detection of pediatric delirium and withdrawal using the SOS-PD is feasible and can identify affected patients outside the PICU. The high number of scoring results per patient-day indicates excellent staff compliance.

In our population of 596 patients, there is a significant number of patients who experience symptoms of withdrawal or delirium (*n* = 94, 15.8%). This is consistent with studies from other high-risk groups in pediatric oncology [15]. By comparing SOS-PD positive and SOS-PD negative patients, we identified potential risk factors for the development of pediatric delirium in a non-critical care setting that are consistent with previous research [2]. Nearly, all affected patients are critically ill and have had a PICU admission. Although young age was a significant factor in our analysis, this association did not reach statistical significance in a recent meta-analysis [2]. Patients with a positive SOS-PD score stayed significantly longer in the PICU, though the absolute difference was minimal. This contrasts with the overall hospital stay, where affected patients experienced a markedly longer duration, highlighting a more substantial clinical impact.

Several validated scoring systems are used to detect pediatric delirium in various age groups, including the Cornell Assessment of Pediatric Delirium (CAPD) and the Pediatric or Preschool Confusion Assessment Method–Intensive Care Unit (p[s]CAM-ICU) [4, 5]. The SOS-PD we used has been validated for most and is used in all pediatric age groups [6]. More importantly, it differentiates between symptoms of withdrawal and delirium [6, 17, 18].

In our cohort, there is a significant overlap between patients who had a positive score for delirium and withdrawal. This is further underlined by the fact that many of our patients still receive analgesia and sedation using morphine, fentanyl, and clonidine in this non-critical care setting. Patients with delirium did not show any difference from patients with withdrawal in respect to age, gender, PICU stay, PICU length of stay, hospital length of stay, or CHD diagnosis (Table 1).

One major modifiable risk factor for pediatric delirium is the choice of sedation [1]. In the PICU, we use a nurse-controlled analgesia and sedation protocol and routinely screen for pediatric delirium and withdrawal [11, 19]. However, outside the PICU, delirium and withdrawal have not been routinely monitored with a structured score before this study. Recognition was dependent on the clinical assessment and experience of healthcare providers. In contrast, even most PICUs do not routinely screen for pediatric delirium, and the implementation of available recommendations for the management of pain, withdrawal, and delirium is inconsistent [2, 8, 13, 14, 20]. Therefore, the incidence of pediatric delirium and withdrawal might be higher in other pediatric cardiology departments.

Since our study includes a high proportion of young infants after cardiac surgery, who are known to be at an especially high risk of developing pediatric delirium, the observed incidence might be higher than in other pediatric wards [3]. Conversely, the incidence of pediatric delirium and withdrawal may be even higher in other cardiology units where structured screening is implemented neither inside nor outside the PICU. However, the findings of our single-center study may not be generalizable to general pediatric wards or institutions with different patient populations, admission patterns, or sedation and weaning protocols.

Given the observed burden in our pediatric cardiology ward, standardized assessment for pediatric delirium and withdrawal outside the PICU should be further investigated in larger multicenter studies before routine implementation is considered.

## Limitations

Although delirium assessment scales are deemed valid and reliable, they have limitations. These tools may lack diagnostic specificity when applied to children with developmental delays. In addition, differentiating between delirium and withdrawal in nonverbal children who may be experiencing pain, anxiety, or loneliness is challenging [13]. However, our high screening coverage demonstrates that the implementation of a structured screening for pediatric delirium and withdrawal in a non-critical care setting is feasible.

We conducted thorough training with nearly all healthcare providers before initiating the study and re-educated new personnel in the appropriate application of the SOS-PD score. However, we did not compare and validate our screening results with a gold standard. This has already been done when the SOS-PD score was first implemented and validated [6]. The score is available in German and several other languages.

Delirium is a clinical diagnosis with a broad differential, including infections, withdrawal, and metabolic disturbances, among others. In our study, interpretation of positive SOS-PD scores and subsequent management decisions, such as adjustment of weaning plans or diagnostic workup, were made by the attending clinicians at the bedside. We did not collect detailed contextual clinical data for each positive screening result. This pragmatic approach, consistent with prior studies, acknowledges the fluctuating nature of delirium and the limitations of single time-point assessments [1, 13]. While the SOS-PD includes items that reflect different behavioral phenotypes, it does not reliably differentiate between hyperactive, hypoactive, or mixed subtypes [6, 18]. Moreover, no validated criteria currently exist for subclassifying delirium types based on SOS-PD scores or any other scoring tool for pediatric delirium. Therefore, delirium subtyping was not attempted in this study but should be addressed in future research.

Our study did not include any non-pharmacological treatment or prevention measures such as early mobility or family-centered care, as this has not been part of the initial training of the healthcare providers. However, it has been shown that simply implementing routine screening for pediatric delirium reduces delirium rates [21].

Patients with a positive SOS-PD score not only received analgesic medication more frequently but also significantly more often required treatment with adjunctive sedative agents, including chloral hydrate, levomepromazine, and melatonin. As these medications are often used to control delirium symptoms, reverse causality cannot be excluded, and we only aim to describe an association, not causality.

Patients with a positive SOS-PD score had significantly more frequent and prolonged PICU admissions than those without (Table 1), supporting its role as a risk factor for delirium and withdrawal. However, it is important to note that only a subset of our cohort (34.4%) had a preceding PICU stay, as the cardiology ward also admits patients directly from the emergency department or for elective procedures. Consequently, while prior PICU exposure and associated sedative medication use may contribute to the risk, they do not account for all cases of delirium or withdrawal observed on the ward. Our study focused on screening feasibility and incidence specifically outside the PICU and therefore included only medication data administered during the pediatric cardiology ward stay.

## Conclusion

Using a validated screening tool, we demonstrate that delirium and withdrawal are common outside the PICU, affecting one out of six patients in a pediatric cardiology ward. Affected patients are often younger, have congenital heart disease, are transferred from the PICU, receive more analgesic medications, and experience longer hospital stays. Nurse-driven using the SOS-PD score is effective and feasible. This warrants further research to enhance strategies for routine detection, prevention, and management of pediatric delirium and withdrawal.

## Data Availability

Research data are not shared. The datasets generated and/or analyzed during the current study are not publicly available due to patient confidentiality and institutional data protection policies but are available from the corresponding author on reasonable request.

## Abbreviations

CHD: Congenital heart disease
IQR: Interquartile range
PICU: Pediatric intensive care unit
REDCap: Research Electronic Data Capture
SD: Standard deviation
SOS-PD: Sophia Observation Scale–Pediatric Delirium
